# Monte Carlo cross-validation for a study with binary outcome and limited sample size

**DOI:** 10.1186/s12911-022-02016-z

**Published:** 2022-10-17

**Authors:** Guogen Shan

**Affiliations:** Department of Biostatistics, University of Florida, Gainesville, FL 32610 USA

**Keywords:** Alzheimer’s disease, Binary outcome, Cross-validation, Machine learning, Monte Carlo cross-validation

## Abstract

Cross-validation (CV) is a resampling approach to evaluate machine learning models when sample size is limited. The number of all possible combinations of folds for the training data, known as CV rounds, are often very small in leave-one-out CV. Alternatively, Monte Carlo cross-validation (MCCV) can be performed with a flexible number of simulations when computational resources are feasible for a study with limited sample size. We conduct extensive simulation studies to compare accuracy between MCCV and CV with the same number of simulations for a study with binary outcome (e.g., disease progression or not). Accuracy of MCCV is generally higher than CV although the gain is small. They have similar performance when sample size is large. Meanwhile, MCCV is going to provide reliable performance metrics as the number of simulations increases. Two real examples are used to illustrate the comparison between MCCV and CV.

## Introduction

Machine learning (ML) methods are increasingly applied to improve diagnostic classification in clinical research [5, 9]. For a study with categorical outcome whose classes are known beforehand, supervised ML methods can be used to predict outcomes for a separate data [18, 31]. Linear discriminant analysis (LDA) finds a linear combination of features that separates two or more classes. Logistic regression is a special case of LDA for a two-class classification problem. The k-nearest neighbors algorithm is a simple and easy method that assumes similar individuals being close to each other. Regression tree starts from root of a tree with all the features as nodes [28, 34, 36]. For every possible route from root to the end of a branch that does not split any further, a classification is made. Ensemble classification and boosting are two techniques to improve weak methods, such as stochastic gradient boosting [7]. Random forest achieves classification via a majority voting from all decision trees [20, 21, 26, 42]. Support vector machine (SVM) finds a decision function that maximizes the margin around the separating hyperplane by developing a mapping from features to classes as a combination of kernels [11]. SVMs are preferable in many researches due to their high accuracy in model prediction [38].

Cross-validation (CV) procedure is traditionally applied to build ML models. To perform CV, data are split into *k* small folds (e.g., $k=10$). The majority of these folds are used as the training data and the remaining folds are the testing data. For leave-one-out CV with 10 folds, there is a total of possible 10 CV rounds that is the number of all possible combinations of folds for the training data. When it is computationally intensive, researchers may only run one round in the model building, which could introduce a significant amount of bias in the model performance metrics. In light of this issue, researchers may run all CV rounds to reduce the estimate bias (e.g., 10 rounds in leave-one-out CV with 10 folds).

Alternatively, one may consider Monte Carlo cross-validation (MCCV) that splits data into two subsets by sampling without replacement. MCCV is a simple and effective approach in data science [37]. Xu et al. [40, 41] compared MCCV with leave-one-out CV for a linear regression model with continuous outcome, and found that MCCV has a better performance than leave-one-out CV with regards to mean squared error of prediction. Shao [37] pointed out that the traditional CV approach is asymptotically inconsistent which is a important statistical property of model selection. When the probability of selecting the model with the best predictive ability goes to 1 as the number of observations goes to $+\infty$. Shao [37] provided the asymptoticl consistent property of MCCV for linear models.

In many clinical studies, dichotomous outcome is the parameter of interest, such as disease progression or not in cancer trials [27, 33, 35], and amyloid-$\beta$ status (either positive or negative) in Alzheimer’s disease (AD) research [2, 22, 29]. Amyloid-$\beta$ is considered as one of the two pathologies for diagnosis of AD [14], and has been the target in many recent AD trials using disease modified therapies (DMTs) (e.g., the BAN2401 trial [15]). One important drug is Aducanumab that is an antibody drug to remove amyloid-$\beta$ plaques for individuals at early stages of AD [16]. In that trial, a positive amyloid Positron Emission Tomography (PET) scan was one of the inclusion criteria. However, PET scan is very expensive and it is often not covered by insurance. Effective ML methods have the potential to save costs and screen proper participants faster.

## Methods

We are going to compare MCCV with CV for a study with binary outcome (e.g., disease progression or not). We use 10-fold leave-two-out CV to build a predictive model by using 8 subsets as the testing data and the remaining 2 subsets as the testing data. There is a total of 45 CV rounds which are the number of combinations choosing 8 folds from a total of 10 folds. To perform MCCV, data are split into a training set (80%) and a testing set (the remaining 20%) without replacement in each simulation. For a fair comparison between MCCV and CV, we use the same number of rounds as that in the CV: 45 simulations in MCCV.

The following 12 supervised ML methods for binary outcome are studied: (1) linear discriminant analysis (LDA); (2) generalized linear model (GLM); (3) logistic regression (LOG); (4) naive bayes (BAY); (5) bagged classification and regression tree (CART); (6) recursive partitioning and regression trees (TREE); (7) k-nearest neighbors (KNN); (8) random forest (RF); (9) learning vector quantization (LVQ); (10, 11) support vector machines with linear kernel (SVM-L) or polynomial kernel (SVM-P); and stochastic gradient boosting (SGB). We use the statistical package *caret* from *R* to implement these ML methods [10, 31], with the detailed function values in Table  1. In the statistical package *caret*, an inner CV with 10-fold is performed on the training set, also known as the nested CV.

**Table 1:** Twelve supervised ML methods from the R package *caret*

Method	ML model	Method value in R
LDA	Linear discriminant analysis	lda
GLM	Generalized linear model	glm
LOG	Boosted logistic regression	LogitBoost
BAY	Naive Bayes	Naive_bayes
CART	Bagged CART	Treebag
TREE	Recursive partitioning and regression trees	rpart
KNN	k-Nearest neighbors	knn
RF	Random forest	rf
LVQ	Learning vector quantization	lvq
SVM-L	Support vector machines with linear kernel	svmLinear
SVM-P	Support vector machines with polynomial kernel	svmPoly
SGB	Stochastic gradient boosting	gbm

Accuracy is one of the most common performance metrics to evaluate ML models, and it is calculated as the proportion of all samples from a testing data that are correctly predicted by using the predictive ML model built from a training data [18]. It is defined as:

$$\begin{aligned} Accuracy=\frac{TP+TN}{TP+TN+FP+FN}, \end{aligned}$$

where TP, FN, TN, and FP are the numbers of true positive, false negative, true negative, and false positive, respectively. It is easy to show that the total sample size is $N=$TP+TN+FP+FN. When comparing different ML methods, the one having the highest accuracy is preferable. In this article, we focus on comparing accuracy between MCCV and CV instead of identifying optimal ML methods.

## Results

We first apply the aforementioned ML methods to predict amyloid-$\beta$ positivity using two data sets: (1) Alzheimer’s Disease Neuroimaging Initiative (ADNI), and (2) Center for Neurodegeneration and Translational Neuroscience (CNTN).

### ADNI data

We first use data from the ADNI [39] to illustrate the application of the considered ML methods to predict amyloid-$\beta$ positivity among individuals with significant memory concern (SMC). Individuals with SMC are at an early stage of dementia, and they become one of the target population in AD clinical trials to alter the disease progression by starting intervention earlier [16]. The SMC group was enrolled during the second phase and the third phase of the ADNI [1]. The ADNI study is a longitudinal study having one of the goals to accelerate the AD drug development by discovering new biomarkers.

In this example, let the outcome be the amyloid-$\beta$ status, which is defined by using a threshold of 1.11 from the computed standardized uptake value ratio (SUVR). The SUVR is an average of weighted four cortical retention means divided by the whole cerebellum SUVR. Four regions are: frontal, cingulate, parietal, and temporal regions [6, 12, 13]. The SUVR value is obtained from the baseline amyloid positron emission tomography (PET) scan.

The following 11 features are used in the ML models. APOE $\varepsilon$4 gene is the well-known genetic risk factor for patients with AD. Additional one copy of APOE $\varepsilon$4 gene would increase the risk of developing AD by 4-fold or more [8]. Six demographic features are: age, sex, race, years of eduction, hispanic ethnicity, and marital status. The neuropsychological scores from the following four tests are also included as features: (1) Clinical Dementia Rating-Sum of Boxes (CDR-SB), (2) Mini Mental State Exam (MMSE), (3) Montreal Cognitive Assessment (MoCA), and (4) the 13-item ADAS-cog (ADAS-cog13). Among these features, many of them are continuous measures, especially the cognitive tests.

The characteristics of the SMC individuals are presented in Table 2. Participates are elderly with the mean age of 71.85, and the majority of the participants are Whites (close to 90%). We also present the Pearson correlation coefficient of each feature with the amyloid-$\beta$ status. The genetic risk factor of AD (APOE $\varepsilon$4) and age are two features having significant correlations with the amyloid-$\beta$ status. The other features do not have strong correlations with the outcome, while the marital status and sex have week correlations with the outcome.

**Table 2:** Patient characteristics of SMC individuals from the ADNI study

	N = 149	$\rho$ (*p* value)
Amyloid-$\beta$ positivity (%)	59 (35%)	
*APOE* $\varepsilon 4$		0.2670 (0.0004)
0 Copy	109 (64.50%)	
1 Copy	54 (31.95%)	
2 Copies	6 (3.55%)	
Age	71.85 (6.11)	0.2255 (0.0032)
Edu	16.83 (2.54)	0.0136 (0.8606)
Sex	99 (58.58%)	$-0.1370$ (0.0756)
Hispanic	6 (3.55%)	$-0.0064$ (0.9347)
*Race*		$-0.0927$ (0.2306)
Whites	152 (89.94%)	
African American	10 (5.92%)	
Other	7 (4.14%)	
*Marry status*		0.1435 (0.0627)
Married	123 (72.78%)	
Never married	10 (5.92%)	
Divorced	18 (10.65 %)	
Widowed	18 (10.65%)	
ADAS-cog13	10.40 (4.53)	0.01239 (0.8734)
MoCA	25.93 (2.65)	$-0.07430$ (0.3370)
CDR-SB	0.06 (0.17)	0.02516 (0.7454)
MMSE	29.07 (1.17)	$-0.05532$ (0.4750)

The last column is the Pearson correlation coefficient between each feature and the outcome (Amyloid-$\beta$)

**Fig. 1:**
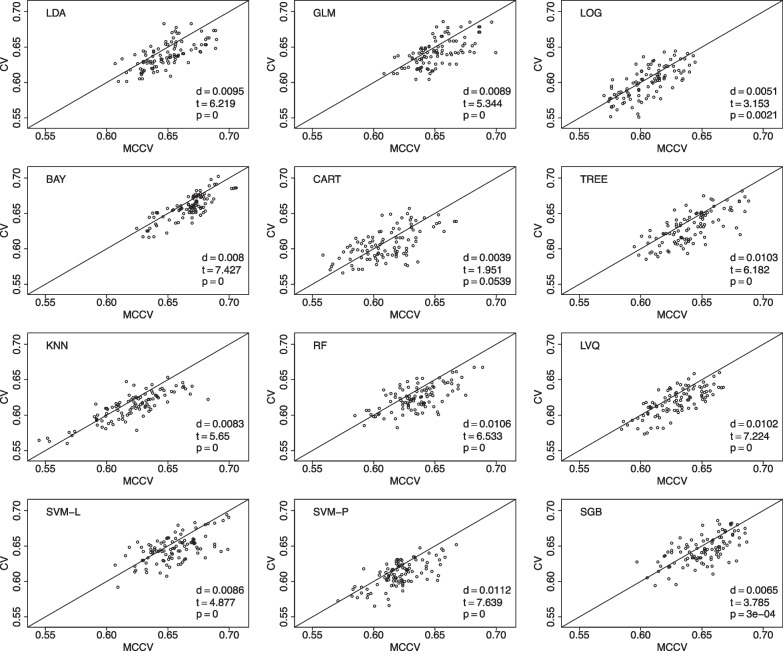
Data from the ADNI is used to compare MCCV and CV of the 12 ML methods with 100 simulations using accuracy as the performance metric in model building. Within each simulation, there are 45 rounds. When *d* is positive, MCCV has a better performance than CV

**Fig. 2:**
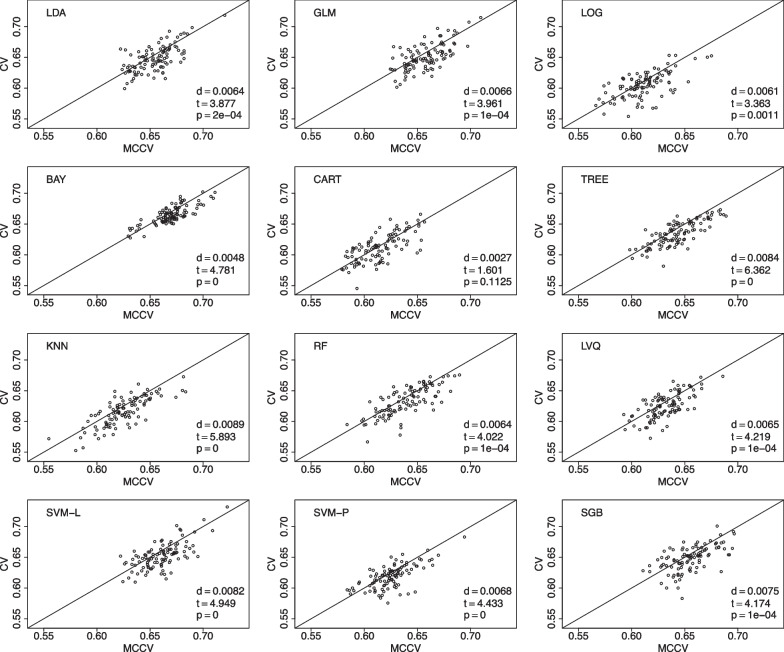
Data from the ADNI is used to compare MCCV and CV of the 12 ML methods with 100 simulations using F1 as the performance metric in model building. Within each simulation, there are 45 rounds. When *d* is positive, MCCV has a better performance than CV

We have 169 individuals with SMC in the database. We choose to randomly pick 150 individuals for 100 times. For each selected data, we follow the aforementioned procedure to randomly split data into 10 folds in leave-two-out CV, with a total of 45 CV rounds. Similarly, we run 45 simulations in MCCV, and compare its average accuracy with the average accuracy in CV. The value of *d* is the mean accuracy difference between MCCV and CV (MCCV-CV), *t* is the test statistics from a paired *t*-test, and *p* is the pvalue from the paired *t*-test to assess the difference between MCCV and CV. When *d* is positive, it suggests that MCCV has a higher average accuracy than CV. In Fig. 1, we find that MCCV always has a higher average accuracy than CV in all the considered ML methods. In addition to accuracy, we also compare these two approaches with F1 score which takes into account of possible mis-classifications. We present the model comparison using F1 score in the model building in Fig. 2. It can be seen that MCCV has a better performance than CV with all the *d* values are positive for all the ML methods.

**Table 3:** Comparison of the 12 ML methods with regards to performance metrics (accuracy and F1) and the statistical test with assumption check by using data from the ADNI study

Method	Normality	Levene test	Paired *t*-test		Accuracy		F1	
			Test statistic	*p* Value	MCCV	CV	MCCV	CV
LDA	0.102	0.962	0.009	0.000	0.649	0.639	0.654	0.648
GLM	0.798	0.443	0.009	0.000	0.651	0.642	0.658	0.651
LOG	0.898	0.129	0.005	0.002	0.606	0.600	0.612	0.605
BAY	0.249	0.457	0.008	0.000	0.667	0.659	0.669	0.665
CART	0.473	0.754	0.004	0.054	0.610	0.606	0.614	0.612
TREE	0.632	0.193	0.010	0.000	0.641	0.630	0.643	0.635
KNN	0.129	0.086	0.008	0.000	0.621	0.612	0.625	0.616
RF	0.733	0.475	0.011	0.000	0.635	0.624	0.639	0.633
LVQ	0.095	0.828	0.010	0.000	0.629	0.619	0.632	0.625
SVM-L	0.591	0.288	0.009	0.000	0.654	0.646	0.661	0.652
SVM-P	0.475	0.435	0.011	0.000	0.621	0.610	0.626	0.619
SGB	0.609	0.946	0.006	0.000	0.651	0.645	0.656	0.648

In Table 3, we present the detailed performance metrics (accuracy and F1) for each ML method. For this data set, it can be seen that MCCV has better performance than CV. The accuracy and F1 values of MCCV are larger than those using CV. The *p* values for normality test based on the Shapiro Wilk’s test and equal variance test based on the Levene test are provided in this table. All these *p* values are above 0.05. We do not have sufficient evidence to the reject the normality assumption and the equal variance assumption. The paired *t*-test is used to compare the accuracy difference between MCCV and CV. All ML methods show that MCCV has a statistically significant higher accuracy than CV. Although not presented here for the paired *t*-test for F1 in this table, the results are similar to the findings using accuracy as seen in Fig. 2.

### CNTN data

**Fig. 3:**
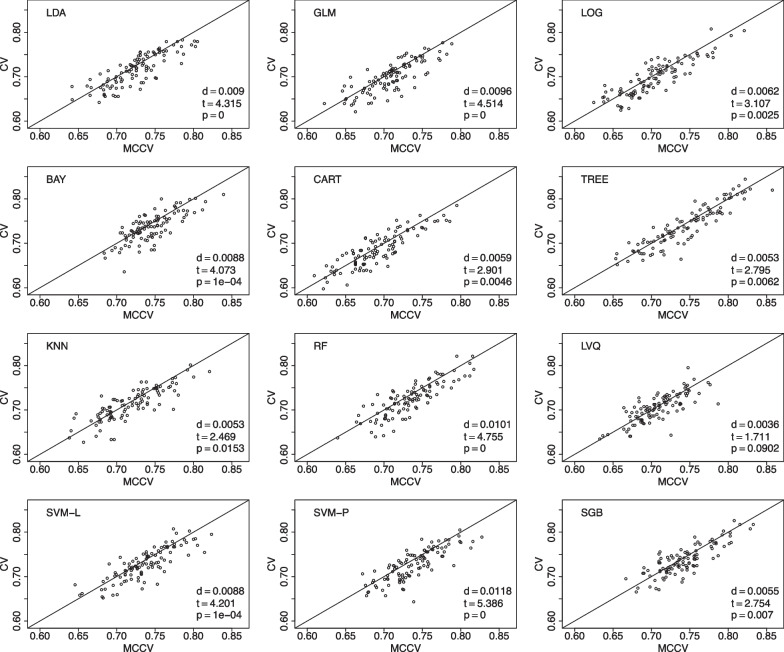
Data from the CNTN study is used to compare MCCV and CV of the 12 ML methods with 100 simulations using accuracy as the performance metric in model building. Within each simulation, there are 45 rounds. When *d* is positive, MCCV has a better performance than CV

**Fig. 4:**
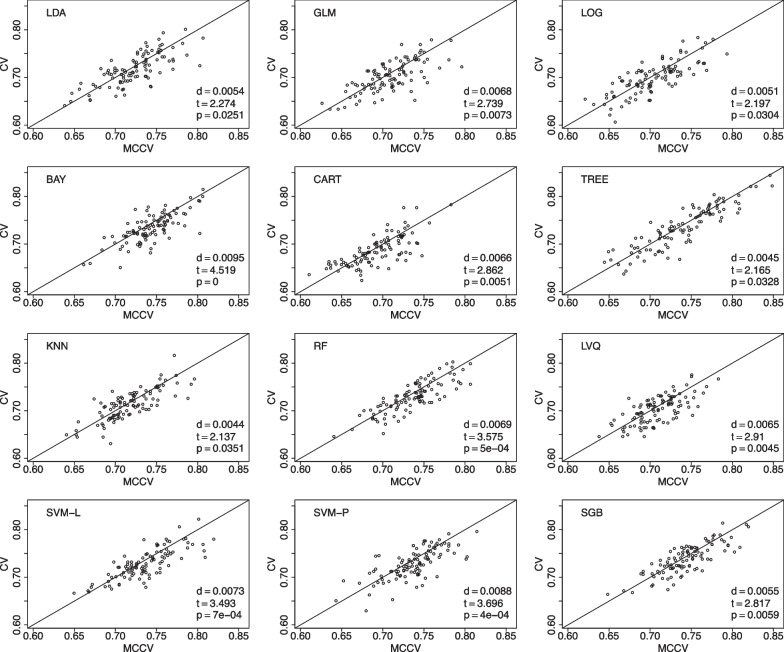
Data from the CNTN study is used to compare MCCV and CV of the 12 ML methods with 100 simulations using F1 as the performance metric in model building. Within each simulation, there are 45 rounds. When *d* is positive, MCCV has a better performance than CV

We use another data from the CNTN study [3, 25] to compare CV and MCCV to predict amyloid-$\beta$ positivity. In this dataset, we have 53 amyloid-$\beta$ positivity cases from the total of 117 participants. The following features are studied: age, race, ethnicity, education, gender, MoCA, MMSE, and CDR. For the last cognitive measures, we only use their total scores. The ML procedure settings are the same as those used in the ADNI example. MCCV is shown to have better performance than CV when accuracy is used as the performance metric in Fig. 3, and F1 score is the performance metric in Fig. 4.

For this example, the time to run all 12 models is 12.6 s using MCCV as compared to 12.1 s with CV using a personal computer. If we run MCCV for 100 time, it will be 21 min. If we run CV only one time, the time would be much shorter, which is 12.1 s. When the total time is not too long, MCCV is recommended.

We conduct the variance analysis for the performance of MCCV and CV. These two methods have similar standard deviation (SD) of their accuracy values. In the ADNI data, the SD values are 0.292 and 0.301 for MCCV and CV respectively, and 0.312 and 0.291 for MCCV and CV in the CNTN data.

### Numeric study

We conduct extensive simulation studies to compare the performance between MCCV and leave-two-out CV. We simulate data from normal distributions, multinomial distributions, and binomial distributions for 20 features, and a binominal distribution for the outcome (*Y*). The first feature ($X_1$) is simulated from $N(0,sd=2)$, a normal distribution with mean of 0 and standard deviation (sd) of 2. The other 11 features follow $N(0,sd=\sigma _k)$, where $\sigma _k$ is a random value from 0.5 to 20, and $k=2, 3, \ldots , 12$. The next four features follow a multinomial distribution with the maximum possible outcome randomly chosen from 3 to 8, and the probability randomly selected from 0.1 to 0.9. The last four features follow a binomial distribution with the probability randomly selected from 0.1 to 0.9. The outcome *Y* is simulated from a binomial distribution with the probability as a function of the first feature: $\frac{\exp ^{\beta _0+\beta _1 X_1}}{1+\exp ^{\beta _0+\beta _1 X_1}}$, where $\beta _0= -1.6$ and $\beta _1=0.1$. The value of $\beta _1$ captures the correlation between *Y* and $X_1$. A high value of $\beta _1$ represents a high correlation.

**Fig. 5:**
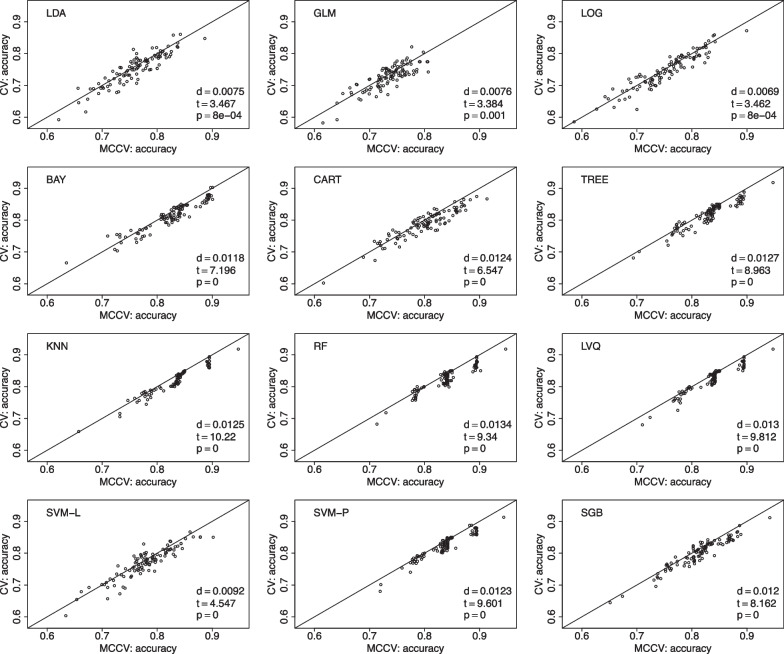
Accuracy between MCCV and CV when $N=100$ and a medium correlation between *Y* and $X_1$ ($\beta =0.1$). When *d* is positive, MCCV has a higher accuracy than CV

**Fig. 6:**
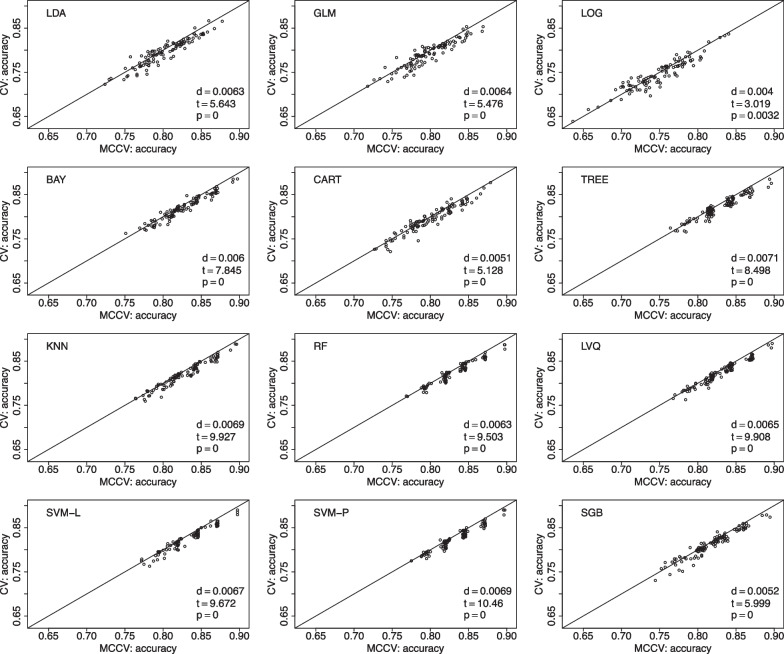
Accuracy between MCCV and CV when $N=200$ and a medium correlation between *Y* and $X_1$ ($\beta =0.1$). When *d* is positive, MCCV has a higher accuracy than CV

**Fig. 7:**
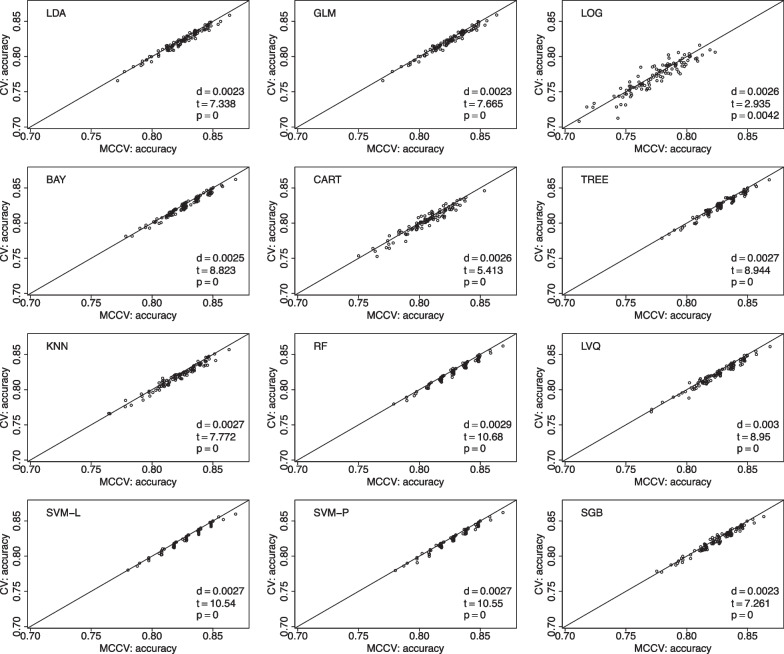
Accuracy between MCCV and CV when $N=500$ and a medium correlation between *Y* and $X_1$ ($\beta =0.1$). When *d* is positive, MCCV has a higher accuracy than CV

**Fig. 8:**
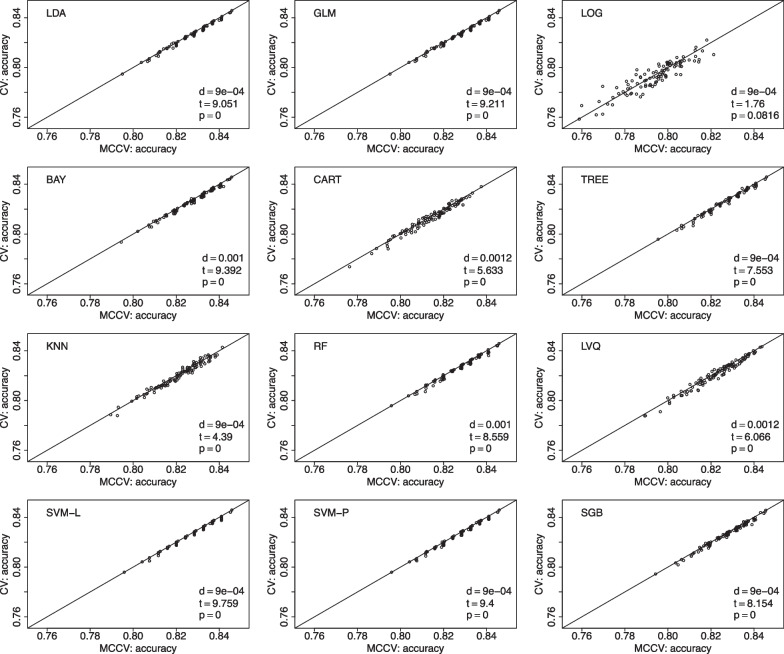
Accuracy between MCCV and CV when $N=1200$ and a medium correlation between *Y* and $X_1$ ($\beta =0.1$). When *d* is positive, MCCV has a higher accuracy than CV

**Fig. 9:**
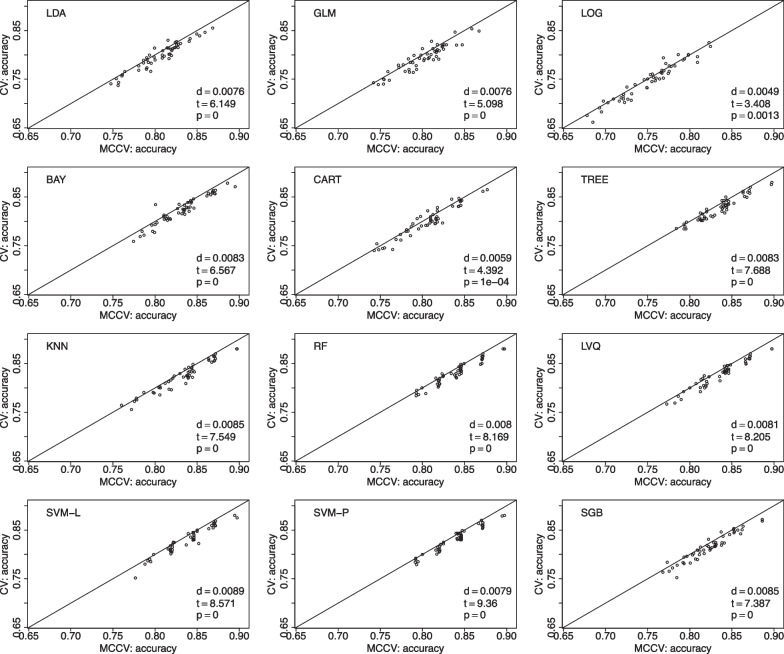
Accuracy between MCCV and CV when $N=200$ and a medium correlation between *Y* and $X_1$ ($\beta =0.05$). When *d* is positive, MCCV has a higher accuracy than CV

We present comparisons between MCCV and leave-two-out CV with 100 simulations when sample size *N* is 100 in Fig. 5. In each simulation, average accuracy is calculated from 45 CV runs as described above. In each plot, there are 100 dots representing average accuracy of MCCV and that of CV from each simulation. It can be seen from the figure that MCCV generally has a higher accuracy than CV. As sample size *N* is increased to 200 (Fig. 6), 500 (Fig. 7), and 1200 (Fig. 8), MCCV outperforms CV when *N* is not very large. In the case when $N=1200$, their accuracies are close to each other although MCCV is slightly better than CV with regards to accuracy. We observe similar results when data are simulated with $\beta _1=0.05$ in Fig. 9 when $N=200$.

When sample size is large, CV and MCCV are almost the same. When a study’s sample size is 500 or below (e.g., Fig. 7), the performance gain using MCCV over CV is still substantial. When each simulation takes very short time, both approaches could be utilized, and MCCV often has better performance than CV.

**Fig. 10:**
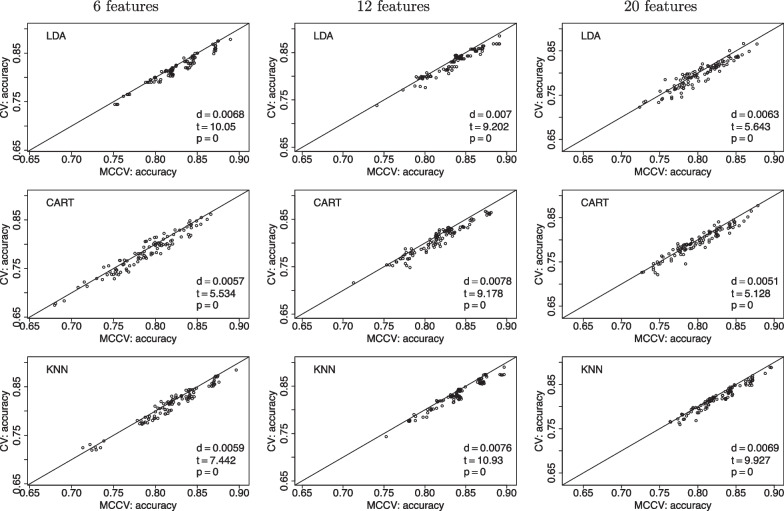
Accuracy as a function of the number of features between MCCV and CV when $N=200$ and a medium correlation between *Y* and $X_1$ ($\beta =0.1$). When *d* is positive, MCCV has a higher accuracy than CV

In Fig. 10, we compare MCCV and CV as the number of features is increased from 6 to 20 for a study with $N=200$. In all the presented cases, MCCV has a higher accuracy than CV with the calculated *d* values being positive. The paired *t* test statistics are relatively large that leads to a small *p* value showing the significant higher accuracy of MCCV as compared to CV. We do not find a clear trend of accuracy as the number of features goes up.

**Fig. 11:**
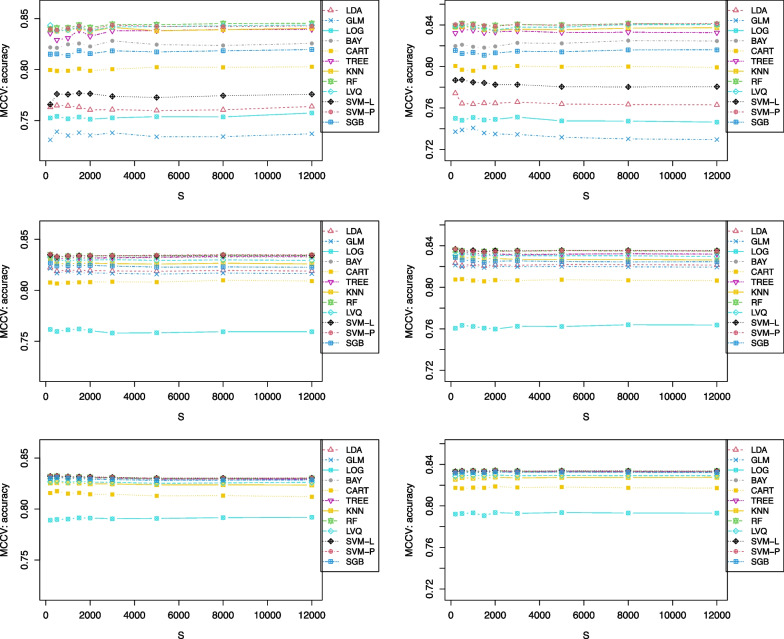
Accuracy as a function of the number of simulations (*S*) for MCCV when N = 100 (top), N = 300 (middle), and N = 800 (bottom), with a medium correlation between *Y* and $X_1$ (left, $\beta =0.1$), and a low correlation (right, $\beta =0.001$)

We also investigate the number of simulations, *S*, needed to have stable accuracy estimates in Fig. 11 with *S* from 200 to 12,000 when sample size *N* = 100, 300, and 800. When *N* is small (e.g., 100), the number of simulations has to be as large as *S* = 5000 in order to have a consistent accuracy. For a study with a large sample size (e.g., 800), it requires fewer simulations to have a stable estimate, such as *S* = 2000.

## Discussion

We compare the performance between MCCV and CV based on popular ML methods when outcome is binary and sample size is limited. In simulation studies, we add a correlation between the outcome and one continuous feature. That pre-specified correlation has some effects on the model prediction. However, ML models are much more complicated than traditionally used statistical models (e.g., logistic regression model) with all features being used in the final predictive model through sophisticated mathematical algorithms (e.g., tree model, SVM). We do not observe a simple relationship between accuracy and that correlation. From simulation studies, we support the findings from this article that MCCV should be recommended for use in practice with a sufficient number of simulations: $S=3000$ when $N<300$ and $S=2000$ when $N\ge 300$.

When *N* is small (e.g., 100), the number of simulations has to be as large as $S=5000$ in order to have a consistent accuracy. For a study with a large sample size (e.g., 800), it requires fewer simulations to have a stable estimate, such as $S=2000$.

Accuracy of ML models is used as the performance metric to compare MCCV with CV. Accuracy is a widely used performance metric in classification problems with known classes of the outcome. Other performance metrics may also be considered, such as sensitivity, specificity, positive predictive value (PPV), negative predictive value (NPV), and the Matthews Correlation Coefficient (MCC). The MCC is equivalent to the Pearson correlation coefficient between the actual outcome and the predicted outcome [19, 23, 24, 32].

For the paired *t*-test to compare the performance of MCCV and CV, we check the normality assumption of the paired data by using their difference based on Shapiro Wilk’s test [30] and the D’Agostino test [4], where the D’Agostino test is a goodness-of-fit measure based on the sample skewness and kurtosis. In a few configurations, the normality assumptions are not met. In such cases, the non-parametric Wilcoxon signed-ranked test may be used to calculate *p* value [17]. For the ADNI example using accuracy as performance metric, the average *p* value is 0.491 with the range from 0.095 to 0.898 for the SW test. The D’Agostino test has the average *p* value of 0.496 with the range from 0.084 to 0.904. The *p* values from the SW test and the D’Agostino test are often close to each other. For the CNTN data, the SW test has the mean *p* value of 0.460 with the range from 0.014 to 0.927. The *p* value of the Wilcoxon test could be slightly larger than that of the *t*-test, but their difference is often very small.

The number of features in ML methods is an important research topic. Xu et al. [41] provided tables for the frequencies of all the possible selected features. When the most relevant features are included in the available features, the performance of MCCV and CV should be similar to their performances with all the features included in the model.

In this article, we split data into 10 folds with 8 folds as the testing data in leave-two-out CV. In the traditional leave-one-out CV approach, we only need to run 10 rounds. With the leave-two-out approach, the number of runs is increased in order to reduce the variance of the model accuracy estimates. When the ML models are not that complicated and data are not extremely unbalanced, leave-t-out approach can be performed, where $3\le t \le 9$. It is also true that leave-t-out CV could be performed when more computational resources are available and sample sizes are large enough.

## Data Availability

Data used in preparation of this article were obtained from the Alzheimer’s Disease Neuroimaging Initiative (ADNI) database (adni.loni.usc.edu), and the Center for Neurodegeneration and Translational Neuroscience (CNTN) database (nevadacntn.org). As such, the investigators within the ADNI and the CNTN contributed to the design and implementation of ADNI and/or provided data but did not participate in analysis or writing of this report.
